# Second Day

**Published:** 1881-06

**Authors:** 


### SECOND DAY—WEDNESDAY, MAY 4, 1881.

The Association was called to order at 10 o'clock by President John T. Hodgen.

The exercises were opened with prayer by Rev. Dr. Peterkin, of St. James' Church.

The Secretary announced the names of the Committee on Nominations. This committee immediately retired to Wilkinson's Hall for organization and for the performance of the work assigned to it.

The Association next considered

### THE SPECIAL ORDER.

Action on amendment to Code of Ethics, Article I, paragraph 1st, add "and hence it is considered derogatory to the interests of the public and honor of the profession, for any physician or teacher to aid in any way the medical teaching or graduation of persons knowing them to be supporters and intended practitioners of some irregular and exclusive system of medicine."

Dr. Cohen, of Philadelphia, called for the reading of the entire paragraph with the amendment.

After the Secretary had read the paragraph with the amendment, Dr. Marcy moved to lay the amendment on the table. The motion was lost—ayes, 74; noes, 76.

Dr. Dunsten, of Michigan, addressed the Association in opposition to the amendment.

Dr. Davis, of Chicago, moved that the amendment be the special order after the addresses to-morrow. Dr. Howard, of Maryland, moved that the matter be indefinitely postponed. The substitute was rejected, and the motion of Dr. Davis adopted.

Dr. John H. Packard, from the Committee on Journalizing Transactions, presented a report, which recommended the establishment of a weekly journal as the organ of the Association.

The report closed with the following resolution:

*Resolved*, That the President be authorized to appoint a committee of five to digest and report in detail as soon as practicable upon the time, place, and terms of the publication of such a journal, to elect an editor, fix his salary, and to arrange all other necessary details.

The report was discussed by Doctors Davis and Toner. Dr. Davis moved to strike out so much of the resolution as related to the election of an editor. Adopted.

Dr. Toner moved that the Secretary and Treasurer be added to the committee. Adopted.

Dr. Toner offered a resolution instructing the Secretary to publish with the forthcoming report of the transactions of the Association, the index of the proceedings of all the previous meetings. Adopted.

REPORT ON NECROLOGY.—Dr. J. M. Toner, from the Committee on Necrology, made the following report:

"In presenting the report of the necrology of the members of the American Medical Association to this meeting, it is but simple justice that I acknowledge the valuable assistance I have received from associates on the committee resident in the several States. While the Society has reason to be grateful for the fidelity with which some of the members have discharged the duties assigned them, there are a few who have totally neglected the obligation they owe to this body, and by such a course defrauded the dead of that respectful notice which this Society has in its wisdom deemed proper, and made provision to give every member who has preserved an interest in it to the end of his life.

Notice in the necrological report of the American Medical Association, in point of honor, may be considered a sort of Westminster Abbey interment or monumental record among the highest medical worthies in the United States, and is a cherished privilege that is assured under the rules to every member of the Association. This right to a notice in the report can only be lost by some unworthy act of the member himself. We should be just to the dead, and every member should feel a personal interest in the matter, and see that a respectable memorial record is neither neglected too long nor entirely omitted. The revised list of all members who at any time attended a meeting of the Association with the years of his attendance given in the volume for 1880, and an alphabetical list which accompanies this report of the names of all the deceased physicians who have had notice taken of their death by this body from its organization, will make the task of performing that duty on the part of the Committee of Necrology in the future an easy one.

"One error I find to be prevalent in the minds of my associates, which is, that members consider it their duty only to prepare notices of those who may die within the year for which they are serving on the committee, while the facts are that all members who have died in good standing, whether in actual affiliation with this body at the time of their demise or not, are legally, under the rules, entitled to notice, no matter how many years have elapsed since their death.

"I trust the index accompanying this report of deceased physicians who have received notices and whose names are scattered throughout its many volumes of transactions, will prove to be of convenience and value in making whatever has been published by us on the subject available. If I may be indulged in the remark, it is my belief that a subject index to all the articles contained in the complete series of our transactions would render them much more valuable to the student and the public than they are at present."

After the announcement of meetings and other notices by the Secretary, the Association adjourned until Thursday at 10 o'clock A. M.

ADDRESS OF DR. WM. PEPPER, CHAIRMAN OF SECTION ON PRACTICAL MEDICINE.—I have found it difficult to comply with the rule providing that the chairmen of the several sections shall read addresses on the advances and discoveries of the past year in the branches of science included in their respective sections. The past year, as regards the progress of practical medicine has been marked, as far as I know, not so much by the announcement of new and important truths, as by the accumulation of a vast number of carefully observed facts of original investigations. These have already been presented to the medical world, in the most accessible forms, in the various abstracts of medical sciences; and to attempt to summarize and reproduce them here will be an undertaking scarcely worthy of the audience I have the honor to address.

It has seemed to me, therefore, that it ought not to be inappropriate to offer a few practical remarks on some one of the medical topics that have occupied most attention of late. It may be safely stated that for some years the largest share of study has been devoted to those great general morbid processes which are grouped under the classes of specific fevers as zymotic diseases. This is not astonishing. The wide prevalence and fatal character of some of these affections demand incessant efforts to detect and remove their causes, to limit their spread, and to modify their course. The enthusiastic zeal with which the study of all pertaining to public health has been taken up of late, and the hopes that are entertained widely, and probably with increasing justification, that this study will lead to a material decrease in the prevalence and fatality of these diseases, fully accounts for the extent to which the minds of medical men are occupied with the subjects of infection, septicæmia, zymosis and its like. The theories upon these subjects have a fascinating interest (the methods of treatment based upon them are often ingenious and plausible), and it is most agreeable to have the mind drawn away from the closeness of the details of individual cases of disease to these broader fields of thought. It is true that there is a wide difference between the amount of fluent writing and talking about septic processes and zymosis and the amount of positive and demonstrable knowledge we have as yet acquired of these recondite matters. The terms and hypotheses that have been devised by investigators to assist them in their efforts to fathom the nature of epidemics have become the common property not only of the rest of us general practitioners, but of the public at large, of the sanitary engineer and of the sanitary plumber; and the subject of blood poisoning has rapidly become one of the fashionable topics of the day.

I speak with no lack of due respect and admiration for the earnest workers on these enormously difficult and important questions, and with no want of appreciation of what has already been done, or of hopeful confidence as to the triumphs of future investigators. But as a general practitioner, I confess that I have been seriously impressed with the danger that follows from the too ready adoption of the popular theory of blood poisoning to explain almost every case of obscure or anomalous character. It will lead to mere scientific expectancy, and to the use of routine methods, to the disregard of that watchful and minute care in adopting remedies to the morbid conditions and the functional derangement present, that constitutes the sole basis of rational and successful practice. Let us consider this important matter somewhat more in detail. In the first place, it is clear that there are some diseases that may be taken as examples of pure specific zymosis, to employ the current term. Typhus fever occurs, to us, as perhaps the best instance of the kind. In uncomplicated cases of typhus fever we have an intense disturbance of general nutrition, without any local lesions of the solids that modify materially the course of the case. The symptoms are the result of the action of a specific cause; no antidote to that poison has yet been found; experience has shown that it will be eliminated from the system in a certain definite time; and therefore it is clear that our treatment resolves itself into the use of carefully adjusted means for supporting the patient's strength until the elimination is completed. If any one symptom attain dangerous prominence, we attempt empirically to moderate it.

Again, it is clear that if we possessed a perfect antidote for the poison of a zymotic disease, our medicinal treatment would resolve itself into the administration of that antidote in suitable doses and forms, with due regard to the state of the digestive organs. It seems as yet presumptuous to hope that we shall ever possess such antidotes for the acute infectious diseases, as we have for syphilis and malaria. But I cannot omit a passing reference to the remarkable results that have recently been observed to follow the use of large doses of bi-chloride of mercury in diphtheria. The original paper that brought this subject to my notice was by Dr. ——, of Alleghany City, and was published in the *Transactions of the Medical Society* of Pennsylvania. The extraordinary statements there made have been confirmed by the experiences, as yet unpublished, of several competent observers. A recent experience of my own was to me so striking that I may be pardoned for alluding briefly to it. I saw last month, with Dr. T. J. Turner of Philadelphia, a child of five years old on the fifth day of a grave diphtheritic croup. Beginning with marked pseudo-membranous deposit in the tonsils, croup had supervened on the third day, and suffocation soon became imminent. Alum emetics secured the discharge of several unusually large thick sheets of membrane, evidently from the whole length of the larynx. But slight relief followed, and on the fifth day it was clear that the membranous exudation occupied the bronchi. She was repeatedly made to breathe the vapor of slaking lime; emetics were given when suffocative symptoms became very urgent; quinia, chlorate of potash and senega were given internally; milk and brandy were given as freely as possible. By the seventh day death seemed unavoidable. Aphonia had existed for five days; nourishment was refused almost entirely; the pulse was over 100, very small and feeble; respirations were seventy, and with shallow gasps. There was marked dullness around the roots of each lung and impaired resonance over both lungs; and the respiratory murmur was scarcely audible and mixed with feeble *râles*. Glandular enlargement had been slight; but the systemic infection was indicated by decided albuminuria, with quite numerous granular epithelial casts. The extremities were cool; the lips livid; the expression indicated advanced asphyxia. Dr. Turner and I agreed we had never seen a patient recover from such a condition; but it was decided to administer bi-chloride of mercury 1-32 grain every second hour, giving in solution in elixir of bismuth and pepsin (as recommended in the paper), each dose also containing 2 drops tr. nux vomica. No other treatment was used. In the next forty-eight hours ½ grain of corrosive sublimate was taken. No movement of the bowels occurred, and not the least irritation of the stomach; on the contrary, a willingness to take nourishment began to reappear; and by the end of that time it was evident that the exudation was softening and that more air was entering the lungs. The same prescription was continued with gradually decreasing frequency for a week, at the end of which time convalescence was fully established; the urine gradually became normal; and the child is now in perfect health.

The extraordinary tolerance of such large doses of bi-chloride of mercury, and the rapid and progressive improvement from so desperate a state, make us regard this as the most remarkable case we have ever witnessed, and will certainly induce us to give further trial to this remedy given in the same manner in cases of grave diphtheritic infection.

The most serious practical injury that seems to me to follow the excessive prevalence of the doctrine of specific self-limited diseases, is the tendency to underestimate the importance of local lesions, and of peculiarities of individual constitutions, as explaining the symptoms and determining the course of diseases. Even in typically specific diseases, as, for instance, in typhoid fever, observation convinces me daily more and more strongly that it is impossible to get the best results from treatment without paying the most careful attention to these questions. There are some local lesions, such as croupous pneumonia, or meningitis, that may appear as complications of specific fevers, which are much less apt to be overlooked than catarrhal lesions of the mucous membranes. Nor is there any danger of being suspected of an attempt to revive the exaggerated but most suggestive doctrines of Broussais by dwelling strongly on the enormous importance of these catarrhal lesions, both as complications of specific diseases and as the essential cause of many cases that are mistaken for specific febrile affections. Rindfleisch certainly does not exaggerate when he says that "the larger part of all the diseases to which humanity is liable, consists of catarrhal affections of mucous membranes, or of disorders complicated by them." In no other country in the world, and among no other people, can this statement be more clearly true than it is here among ourselves. Our physical characteristics and the conditions of the American climate, or climates render this incontestable. But to realize this truth, it is necessary to consider the catarrhal affections in their broadest sense. The conditions that attend acute catarrh are congestion and swelling of the mucous membrane; enlargement of its lymphatic follicles; increase of and changes in its secretions. It is indeed difficult to appreciate clinically more than the last of these conditions, and the term "catarrh" applies strictly only to it. But while in many cases the secretions are excessive, as in cholera morbus, or dysentery, or acute bronchitis, and constitute the most prominent symptoms, there are many cases of catarrhal inflammation where the secretions are perverted but not excessive, so that the presence of the lesion cannot be recognized by any so-called catarrhal discharges. These are cases where, owing to individual peculiarity, or to the localization of the irritation upon some special anatomical elements of the mucous membrane, the amount of muco-serous discharge bears no proportion to the amount of epithelial proliferation, or grandular swelling. One of the best illustrations of this is the cellular catarrh of the lung, a circumscribed catarrhal pneumonia, often involving small areas near the roots at the apex, or indeed in any part of the organ. It is an excessively frequent affection, and I have no hesitation in expressing my belief that owing to the frequency with which it is overlooked, it constitutes the most common starting point of that most common form of consumption, catarrhal phthisis.

There may be scarcely any expectoration, and a cough that is not very annoying, and that may readily be regarded as merely nervous or irritative. Yet the capacity of localized catarrh to induce marked symptoms of constitutional disturbance, is admirably shown in these cases. There will very often be observed a distinct febrile movement of an irregular and often of a distinctly intermittent character; and in consequence of the want of symptoms of marked pulmonary irritation, such cases are continually being mistaken for malarial fever, and being treated with so little rigor that, although the fever subsides, the local lesion is left smouldering till renewed congestion awakens fresh activity, and till slow cheesy transformation occurs and paves the way by septic absorption to the development of tuberculosis. Nothing but an unvarying practice of thorough physical exploration of the lungs in every case of acute disease will obviate these fatal oversights; and I have no doubt that the extreme readiness with which it is customary to regard irregular febrile attacks as malarial, has contributed powerfully to prevent this critical search for slight local lesions in such cases.

The fact that circumscribed catarrhal irritation of a mucous membrane, unattended with any considerable amount of catarrhal discharge, is able to excite marked febrile action, seems to me a matter of the greatest practical importance.

The theory that increased tissue metamorphosis and chemical interchange are the essential causes of and commensurate with the elevation of temperature in febrile diseases, has been the prevalent one for some years past. According to this, it is easy to explain the fever that attends any inflammatory lesion of considerable magnitude. But practically it has seemed to me to be the custom, if some inflammatory center of adequate extent could not be detected, to suspect that the fever was a specific one, due to the admission of an infectious principle into the system, and blood-poisoning, and that the increased chemical changes in the blood were the cause of the febrile movement. This is merely another illustration of the present exaggerated tendency to regard diseases as specific, to which allusion has already been made; and in the hands of certain investigators, chiefly of the German school, whose writings have been so indefatigably and indiscriminately thrust on the mass of American medical students, it has led to methods of practice that appear to me superficial or irrational and mischievous. The degree of fever heat has become a definite entity, to be determined of course by repeated thermometric observations, and to be accepted as dictating absolutely the line of practice. The system is to be supported by abundant food and free stimulation, and the fever reduced by measures proportionate in vigor to its intensity, and if these two indications can be successfully complied with till the self-limited stadium of the disease has expired, treatment has been successful. It matters little which may be the particular antipyretic method that is associated with each degree of fever heat, whether the patient is to have a wet pack or a cold bath so soon as his temperature reaches 103° or 104°, whether these may be postponed till it reaches 104°, that against which clinical experience protests is the one-sided and arbitrary nature of all such rules. We owe very much to recent observers, among whom Walton and Witherle, of our own country, rank prominently, for reasserting the complex and valid nature of fever, and thus furnishing a scientific basis more in accordance with enlightened clinical observation. But it is chiefly from the elaborate and masterly experiments and reasoning of H. C. Wood, whose studies on the pathology of fever, have just been published by the Smithsonian Institute, and constitute in my judgment by far the most valuable contribution of the past year to scientific medicine.

It is impossible to assert that the positions assumed in this memoir have all been demonstrated as yet; but I think it will be generally admitted that it has been shown that the degree of bodily temperature in fever depends, in greater or less measure, upon a disturbance in the natural play between the functions of heat production and heat dissipation, and is not an accurate measure of the intensity of increased chemical movements of the tissues." (Wood, op. cit.) It further appears altogether probaable that an inhibitory thermic center exists, and that this may be so depressed by morbid influences that tissue changes and heat production are increased; while at the same time, owing to the disturbance of the vaso-motor centers, the dissipation of heat is reduced. Some such complex mechanism, involving essentially the co-operation of the nervous system, certainly seems necessary to account for the peculiarities that fever presents in different individual cases of disease, and which call for more rational treatment than is recommended in most of the current writings on febrile diseases. Not only may the depression of the inhibitory center be caused by the circulation in the blood of special morbid matters, but it seems clear that reflex irritation from some centers of local disease may be either so intense or so prolonged as to produce the same result. Moreover, it seems clear that such great differences exist between different individuals in the assisting power of the nerve centres in this respect, as in all others, that very different degrees of fever may result from an equal amount of local disease, or from an equal amount of septicæmia, and that the signification and therapeutic indication of given degrees of fever must vary correspondingly. In a case of typhoid fever, for instance, the temperature rises by the eighth day to 103° or 104°, and the pulse to 110 or 120 deg. If this be the result of a severe zymosis with comparatively slight catarrhal irritation of the gastro-intestinal mucous membrane, and the subjects enjoy good nervous tone, large doses of quinine or salicylic acid, full amount of suitable diet and stimulants, will doubtless be appropriate treatment: or if these fail and the temperature continue to rise, the use of cold water externally is desirable.

Broussais, who wrote his classical work on Chronic Inflammation, before the distinction between typhoid and typhus fever was recognized, and whose practical experience was almost exclusively with typhoid, naturally fell into the error of regarding lesions of the intestinal mucous membrane as essential parts of all fevers; and his judicious principle of treatment was carried to an extreme by his zealous followers, so that the restricted diet and inert medication that was recommended in febrile diseases justly secured for Broussaisism, as it was termed, its rejection by the succeeding generation. But his own teachings were not thus extreme, and they embodied a truth which has of late years been too much lost sight of under the influence of the modern theories of zymosis.

It was not uncommon fifteen years ago to see the steadily increasing aggravation of these symptoms, attended with failure of digestive power, diarrhœa and tympanites, as steadily pursued by progressive increase along the whole line of treatment in the amount and frequency of the doses of quinine, turpentine, carbonate of ammonia, alcohol, milk, and beef-tea, until finally scarce 30 minutes were allowed to intervene between the administration of some powerful drug or concentrated food.

But typhus fever stands pretty much alone in its freedom from local lesions or complications, so that the above theory of treatment is applicable to other zymotic diseases only with considerable modifications.

But in other instances I have found a temperature of 104° to 105° reached daily for 10 or 12 days in cases of typhus fever, where the nervous, circulatory and digestive symptoms all indicated that the septicæmia and the gastro-intestinal irritation were both mild, so that the high temperature was apparently due to a predisposition on the part of the nervous system to a failure of its heat-controlling function. In such cases no serious harm has resulted from the sustained high temperature, but a simple plan of treatment, with scrupulous care to allay the intestinal irritation as much as possible, and the avoidance of any other measures to reduce the temperature, has been followed by satisfactory recovery.

It is not to be wondered at that the exhausted nervous system should become paralyzed by the intense reflex irritation from the inflamed mucous membrane, aided by the poisoning of the blood that must certainly have been increased by filling the intestines with organic matter that could not be digested, but must have putrefied and furnished the most fertile soil for the continued development of fresh specific typhoid poison.

So fully am I impressed with the important part played by the gastro-intestinal mucous membrane in causing or aggravating the symptoms of fever and other diseases, that I am convinced actual harm is often done by the use of remedies calculated to reduce temperature when due solely to the blood-poisoning, but which are too irritating to be tolerated by a morbidly sensitive mucous surface. Undoubtedly the more general introduction of the external application of water as a means of reducing excessive pyrexia has done much good, both directly and indirectly, by checking the use of less efficient and more dangerous antipyretics. But I am satisfied that if the diet and treatment in the early stage of typhoid fever are carefully adapted to the degree of gastro-intestinal catarrh present, the need of cool baths or other extreme means for relieving hyperpyrexia will be comparatively infrequent.

This is not the time to enter into a more full discussion of the management of this interesting disease. So many different remedies and special methods have been vaunted, and their results attested by formidable statistics, that we may well marvel that there is any longer room for difference of opinion on the subject. But it seems to me that so long as this or any other general disease is regarded as a specific entity and treated by any one special method, we shall never learn what is the lowest rate of mortality attainable. This result will be secured in regard to typhoid fever only when we employ a rational method adapted to the complex condition present and take cognizance of the peculiarities of the individual constitution, of the important local lesions, as well as of the blood-poisoning. If it is true that even in undoubtedly zymotic diseases the state of the mucous membrane, especially of the gastro-intestinal tract, has much to do with the production of the febrile symptoms, it is not surprising that we meet with many cases of typhoid fever that are dependent solely upon catarrhal irritation of this mucous surface. This catarrhal fever has been the subject of several well-considered articles during the past year, but I cannot believe that it is as well known as the frequency of its occurrence deserves. It arises from the ordinary causes of catarrhal inflammation, of which atmospheric conditions and changes are by far the most common, and runs a course resembling in many respects that of a mild typhoid fever. The duration varies from nine to twenty-one days; the febrile movement presents marked daily fluctuations; the tongue is coated, the abdomen rather full; the bowels occasionally loose but more frequently quiet, though laxatives will provoke thin, light colored, unhealthy stools; appetite is lost and prostration is marked. Less commonly these may be associated with nasal catarrh with or without epistaxis, or bronchial catarrh.

The nervous symptoms are usually very mild, although in nervous subjects the irritation of the nerves of the gastro-intestinal mucous membrane may excite quite severe reflex nervous disturbances. The course of this form of fever may be greatly modified by treatment and diet, and it is not impossible that the statistics of typhoid fever are often vitiated by including a greater or less proportion of these simple catarrhal cases. The lesions must be of a moderate grade only, rarely going on to ulceration, since the prognosis is uniformly favorable. It is possible, however, by the continuance of bad hygienic influences, or by the use of too much and too strong food, and of free stimulation and of irritant remedies, to aggravate the catarrhal irritation, and that ulceration will ensue; the secretions will become more and more vitiated, and finally a secondary infection of the system may be established with grave symptoms of septicæmia.

Lastly, it is interesting to note the important *rôle* that catarrh of the gastro-intestinal mucous membrane plays in connection with many inflammations of other organs. For instance, in pneumonia, the morbid influence that has caused the pulmonary influx, frequently causes a catarrh of the mucous membrane that is really a distinct pathological condition, although it is customary to include its results among the symptoms of the major disease. Practically, however, it demands that our treatment shall be adapted to the complex condition present, and any plan of treatment that addresses itself solely to the process in the lung must often prove ineffectual if not mischievous. No one who has witnessed the excellent results of prompt treatment in acute pneumonia—including suitable depletion or counter-irritation, according to the grade of the disease and the constitution of the individual; carefully restricted diet; absolute rest; sedatives and resolvents given with a critical regard for the state of the gastro-intestinal mucous membrane; and quinia, given if need be by the rectum, so as to avoid increasing gastro-irritation—will hesitate long before accepting the theory that this is a specific, self-limited fever, the symptoms of which are the necessary results of the evolution of a special poison in the blood. When we reflect upon the policy of inaction that is wont to follow upon the adoption of such a theory in regard to pneumonia, to rheumatism, or to dysentery; upon the disregard of important local symptoms as being wholly subordinate to the hypothetical blood-poison; upon the tendency to direct treatment solely to the reduction of the elevated temperature without due regard to the complete mechanism and varying significance of such elevation, and by means that may at times react unfavorably on the organic conditions present; it seems to me full time that a halt should be cried to the rapid spread of such speculative doctrines, which are open to severe criticism in theory, and which certainly seem opposed to the results of extended practical experience.

Important as are the varying expressions and relations of catarrhal inflammation in its acute forms, fully as great interest attaches to the study of chronic catarrhs. Close study of the cause of very many chronic diseases will convince us that they do not depend on one continuous morbid process, pursuing a slow and necessary course; but that the affected part, having had its power of resistance reduced by the original inflammatory attack, or by a series of congestions, has finally become the seat of chronic catarrhal changes, that are continually prolonged by the recurrence of fresh attacks upon more and more trifling provocations. This history has constant illustration from every mucous surface of the body; and the fuller clinical study of these chronic catarrhal processes, in the light of our modern pathology, would be replete with interest.

It must suffice here to allude to the remarkable results produced in persons of sensitive, nervous temperament by long-continued catarrhs, even of small extent. The prolonged local irritation ends by causing a morbid reflex excitability of the nerve centers with exhaustion of nerve power such as we are so painfully familiar with in gleet, in chronic uterine catarrh, in nervous dyspepsia. It is often one of the most difficult problems to decide, and upon the decision must depend the method of treatment to be pursued, whether an overtaxed or depressed state of the nervous system has been the initial trouble and has led to the impaired function of the stomach or uterus; or whether the long-continued local disease has induced the nervous disturbances. All are familiar with both of these relations; and the powerful effects produced on the nerve centers by prolonged local catarrhs serves to illustrate the important part that is undoubtedly played by acute catarrhal irritation of the gastro-intestinal mucous membrane in the production of the nervous symptoms of many inflammatory and zymotic diseases.

The injurious effects of chronic catarrh are of course most marked when, as in the case of catarrhs of the alimentary tract, they lead to mal-assimilation of food and consequent depravations of the blood. It is not necessary that such catarrhs should be attended with excessive secretion that would constitute a direct drain upon the system. There may be a single partially soft stool daily; or more commonly there may be a single undigested stool once in two or three days, and during the intervals entire quiet of the bowels; (or finally there may be habitual constipation); and yet all the while a slow catarrhal process may be present in the epithelial layers and lymphatic follicles of the intestinal mucous membrane. In many cases of so-called nervous prostration, in many cases of causeless insomnia, the essential cause of the morbid condition will be found on close search to be a localized catarrh of some part of the alimentary tract, the cure of which by suitable diet, hygiene and drugs, will be speedily followed by restoration to health.

A second and sufficiently common result of chronic catarrh is the maintenance of a slow irritative fever. Allusion has already been made to the irregular, or intermittent fever that often attends acute alveolar catarrh of the lung, or acute catarrh of the gastro-intestinal mucous membrane, and which may readily be mistaken for malarial fever. I have not infrequently met with cases where an obstinate irregular febrile movement, which was regarded as the result of chronic malaria, but which had entirely failed to yield to antiperiodics, was in reality dependent on a gastro-hepatic or gastro-intestinal catarrh. This observation must be a familiar one to all who live in malarial districts; and yet I suspect that the importance of such catarrh, either as a complication of malarial attacks, or as causing a fever-simulating malaria, is hardly sufficiently recognized. It is not necessary to assume that in such cases, where catarrhal inflammation maintains an irregular fever, there is produced any septic matter to be absorbed, and to assist the poison through its action on the blood, or on the nervous system. It seems probable that the recent investigations as to the mechanism of fever will lead to a more simple explanation through the direct agency of the nervous system.

Allusion must finally be made to another and the most serious manner in which chronic catarrh influences the system to produce constitutional diseases. When the catarrhal swelling of the mucous membrane of a duct, as of the common bile duct, imprisons secretions rich in organic matters, decomposition is apt to occur, and poisonous matters are formed and absorbed, which give rise to serious septic fever, such as that with which we are familiar under the name of "hepatic" fever in the above-named pathological condition. More serious still are the consequences that arise when, owing to the anatomical arrangement of the lung, the catarrhal products themselves are retained, and undergo slow, cheesy degeneration. The enormous frequency of alveolar catarrh, the conclusive evidence of its tendency to terminate in this way, and the demonstration of the connection between softening cheesy deposits and infection and tuberculosis of the surrounding tissues and of the system at large, are facts that give to this process a supreme practical interest and importance.

If in other diseases the tendency of the day to overlook the local lesion, and to assume the existence of a specific, self-limited constitutional infection or affection, is to be deprecated, far more dangerous and prejudicial is such an unwarranted assumption in regard to that large proportion of cases of phthisis which originate in catarrhal processes. There are undoubtedly rare cases of primary tuberculosis; there are also many cases where the constitutional tendency to the acquisition of tuberculosis is so strong that it is impossible to prevent its development from the most trifling provocation; there are still other cases where the first attack (of catarrhal pneumonia for instance) causes irreparable injury and leads inevitably to destructive phthisis; but my own experience points clearly to the fact I have already stated, that in the great majority of cases the disease begins as an acute circumscribed catarrh which, from inedequate treatment, from inherent weakness of tissue, or from neglect of hygienic rules so that similar attacks are allowed to recur and recur, pave the way for a chronic catarrhal process of which cheesy degeneration and ulcerative softening, with or without tuberculous infection, are the common and fatal consequences.

I can draw no other conclusion from careful and extended clinical observation than that in its early stages this, the commonest form of phthisis, can be largely controlled by treatment embracing hygienic, dietetic and medical means assiduously and persistently employed. It is of course evident that of any given number of such patients who are placed under fair hygienic conditions and trusted to the influences of nature alone, some proportion will recover, because, in them, after the removal of the depressing influences that have reduced the tone of the system and rendered it susceptible to catarrhal attacks, the tissues retain sufficient vitality to react and to prevent the extension of the local disease, and because no special tendency exists to tuberculous infection. But it seems equally evident to me that this proportion is capable of being greatly enlarged, and the duration of the disease greatly reduced, by the persistent employment of skillful and appropriate treatment. And further, it seems clear to me that to attempt to establish a theory that phthisis is a self-limited disease, in any special sense of the term, is to ignore the teachings of modern pathology and the results of clinical observation.

I have been led much further in these remarks than I proposed to go, but I have been drawn on by the sense of the great practical importance, of a clearer recognition of the large part played by local catarrhal inflammation, in the causation of symptoms now too readily attributed to some hypothetical specific or zymotic process. I do not feel at all satisfied with the theories of treatment largely based upon these hypotheses, that are found in the works on medical practice, for the most part translated from foreign languages, which burden our shelves. I am sure they do not accord with the sound practical experience of American physicians, and do not meet the conditions of disease with which we are familiar. I am hopeful that ere long a truly national American system of medical thought, and teaching will be developed, which shall be animated by a more practical spirit, and shall embody the results of the broadest and most thorough clinical work.

ADDRESS OF DR. J. R. CHADWICK, Chairman of Section on *Obstetrics and Diseases of Women and Children.*—Instead of the custom of presenting in this address the advances that have been made in the department of Obstetrics and Gynæcology during the past year only, I propose to ask your attention to a statistical consideration of the whole volume of literature upon this branch of medicine during the past five years. I shall present a quantitative analysis of this literature with reference to nationalities, and shall finally sketch the rise and fall of the interest evinced in certain special topics, as shown by the number of separate publications and journal articles published in each successive year. These enumerations I am enabled to make by utilizing the annual "Indices of the Gynæcological and Obstetric Literature of all Countries," published under my supervision in the "Transactions of the American Gynæcological Society." The index for the year 1876 only covers the last six months of the year, and is therefore of but limited utility. The Index for the year 1879 is in print, but is not yet published; that for 1880, though, in form of copy, now in course of preparation for the printer, has nevertheless proved available for my purpose. The space of time covered by my review is unfortunately limited to five, or, more strictly speaking, to four and a half years, so that my sketches of the different topics can in no instance embrace more than a brief epoch in the life-history of any method of treatment, operation or theory. Such a numerical analysis of the current literature, if it could be made to cover a longer term of years, would, I am convinced, show some strange mutations in the popularity of certain topics, and would dampen the ardor of many authors who have sought to attain immortality by propounding new theories, devising new operations, and above all, by inventing new instruments. Of course the number of articles published upon a given new topic is not, in every instance, an accurate test of its merit. Many new ideas are of such nature as to appeal to a limited audience. A new therapeutic remedy or a new operation, on the other hand, is pretty sure to meet a warm reception. Its life-history may then be outlined somewhat as follows: An article is written, recounting the success obtained by its author in the treatment of a certain condition by a new operative method. Immediately it is tried by many practitioners, who hasten to publish their results, particularly if favorable, when they may expect to derive renown or practice from being early identified with the innovation. Articles multiply rapidly, the operation has been forced upon the attention of the whole profession; soon its charm of novelty wears off, and the number of papers would rapidly diminish were it not that the negative or unfavorable results obtained begin to be published; the true merits of the operation are gradually reached and finally it is either adopted as part of the traditional story of operative procedures or is renounced and forgotten. A familiar recent instance of this course of events is observable with regard to incision of the cervix uteri for dysmenorrhœa advocated many years ago by Sir James Simpson. This operation at once appealed to the mechanical instincts of the profession, was taken up with avidity, was advocated in all the medical journals of the world, was variously modified by different operators, each of whom sought by numerous papers to have his special method adopted; opponents soon raised their voices; the operation was shown conclusively not to be followed by the promised relief in many cases, and moreover to be fraught with some danger. To-day this operation is rapidly passing into oblivion. It is still performed in cases of extreme stenosis, but is recognized as inadequate to afford relief in the vast majority of cases for which it was formerly recommended. My table shows a progressive decrease in the number of papers on this subject during the past five years. A similar fate may safely be predicted for several operations now held in high esteem such as the operations for lacerations of the cervix uteri, Freund's method for the complete extirpation of cancerous uterus, Pond's Cæsarean section followed by excision of the uterus, etc.

*Obstetric and Gynæcological Periodicals.*—This table shows that the total number of periodicals has increased in the five years from 13 to 22, nearly 60 per cent.; that the American have increased 100 per cent.; the French, 50 per cent.; the German, 30 per cent.; that Belgium, Italy and Denmark have entered the lists with one periodical each; and that England and Spain have remained unchanged. [This table is omitted.]

These special periodicals may be regarded as one gauge of the degree of interest taken in this branch of medicine in the different countries.

Certain characteristics distinguish the publications of the different countries. For instance, those of Germany contain more contributions to the science of medicine; whereas those of America contain more papers of a practical nature; those of France are more controversial, being more essentially the organs of separate cliques.

Another means of estimating the relative prominence of this branch of medicine in different countries is an enumeration of the special societies. [This list is omitted.]

We see here at a glance how greatly America predominates in this list, containing as many societies as exist in the rest of the world. Too great significance must not, however, be attached to this fact, for one cause of this development of societies is purely geographical. In England it is possible for those who are specially interested in gynæcology and obstetrics to attend the meetings of the Obstetrical Society at London, as actually happens, whereas in America the distances to be traversed are so great as to render this impossible. As a result of these two extremes, we find the English Society has 300–400 members, while the membership of the American societies ranges from 20 to 50. The lack of any special society in France is striking, and unaccountable when contrasted with the large number (6) of special journals issued in that country.

We now come to the consideration of the whole bulk of the special literature; it may best be appreciated by the aid of this table:

| Last six months of— | Sep. Pub. | | Articles. | | Articles. | | Total. |
|---|---|---|---|---|---|---|---|
| 1876 | 91 | = | 1374 | + | 753 | = | 2127 |
| 1877 | 194 | = | 2388 | + | 1698 | = | 4086 |
| 1878 | 168 | = | 1320 | + | 2203 | = | 3523 |
| 1879 | 265 | = | 2194 | + | 2644 | = | 4838 |
| 1880 | 178 | = | 2166 | + | 2614 | = | 4729 |

In this table I have resolved the separate publications into their equivalents in journal articles reckoning an article at an assumed average length of ten pages, which is certainly above, rather than below, the average length of articles. The rate will, however, serve as a convenient basis for the reduction.

It will be noticed in the first place that the volume of this special literature is increasing at the rate of 25 per cent. a year. The figures for 1880 are undoubtedly too small, for the reasons that the titles are not all at hand at this date. I expect to add to these aggregates before this paper goes to press. The volume of literature thus shown to be devoted yearly to a single branch of medicine is certainly appalling, and fully justifies the urgent need for specialization of practice already in process of evolution. Even admitting that 90 per cent. of these writings merely reiterate facts in medicine already familiar to gynæcologists, yet there still remains an immense amount of reading incumbent upon one who would keep abreast with the advances in this, to say nothing of the collateral branches of medicine.

A still further analysis of these figures for one year (1880) discloses some interesting facts with reference to nationalities.

In the first place, France has issued the greatest volume of literature, equivalent to 1,643 articles; America, 1,319; Germany, 669; Great Britain and Ireland, 488.

Dividing these into general subjects, we have:

France—Obstetrics, 941; Gynæcology, 702.

America—Obstetrics, 608; Gynæcology, 708.

Germany—Obstetrics, 350; Gynæcology, 669.

Great Britain and Ireland—Obstetrics, 231; Gynæcology, 257.

This predominant interest of France in obstetrics, may be shown to be restricted to a moderate number of writers, for in analyzing the French contributions closely, it appears that 51 separate publications (books, theses, etc.) were published during the year, which had been estimated as equivalent to 598 articles; deducting these from 941, we have remaining only 343 real germinal articles. In America only 8 books were issued, equivalent to 113 articles, leaving 485 articles. In Germany, a similar process reduced the articles to 159, in England to 158.

Under Gynæcology similar reductions change the relations as follows:

France, 293 articles.

America, 346 articles.

Germany, 201 articles.

Great Britain and Ireland, 162 articles.

The inferences from these figures may be summed up as follows: In America there is a much greater number of contributions in both general branches than in any other country.

In France the whole volume of obstetric literature, nevertheless, exceeds that of America by one-fifth, a curious discrepancy which may be attributed mainly to two factors: first, the publication of graduation theses; and second, the stimulus given to the professors to the writing of treatises from the rivalry engendered by the close propinquity of the fields of their labor; *i. e.*, by their being mostly inhabitants of Paris, and having charge of competing clinics. The cause of the small number of contributions from Germany is probably due to the fact that they emanate chiefly from university professors and instructors whose chief motive is the desire for academic promotion, and whose writings are consequently, for the most part, records of extensive research or investigation; they are, hence, much longer on the average, and require a longer time for their preparation. I am at a loss to explain the lack of interest displayed in England by these figures.

Carrying my analysis one step farther I shall finally bid you scrutinize a table in which are given the number of articles upon certain definite topics in the successive years, and shall offer a few suggestions in explanation.

Under the general heading of ovariotomy we see a marked increase from year to year until a falling off is recorded for 1880. Under the sub-head of "ovariotomy" there is a steady increase, while in the past two years there has been a steady decrease in the number of "cases of ovariotomy;" the change must be apparent to all.

"Normal ovariotomy," which was devised and first carried out by the genius and pluck of our countryman, Dr. Battey, has deservedly claimed more attention during the past three years than any other new operation; but during 1880 its volume of literature has been on the wane, its true value and limits of usefulness having been nearly defined.

Porro's Cæsarean section with extirpation of the uterus appeared as a new operation in 1879, and has since been vigorously advocated and discussed.

The central and systemic effects of renal disease recorded under several headings in the table [omitted] show in the aggregate a marked increase in prominence quite commensurate with their importance.

The "forceps" were brought forward as a special topic in 1878 by the invention of new form by Tarnier, and in 1879 by the formal discussion of the extent of their utility at the Obstetrical Society of London; hence the greater figures under these two years.

"Tumors of the uterus" continue to attract an interest proportionate to the extension of abdominal surgery.

"Extra-uterine pregnancy" has been brought to the front during the past five years mainly through the labors of Parry and Thomas, both Americans.

"Puerperal septicæmia" evinces a small but increasing popularity commensurate with interest now taken in all diseases attributable to putrefactive changes or the development of germs.

"Cancer of the uterus" received an impulse in 1879, which is more than sustained in 1880 by the publication of the alleged curative effect of Chian turpentine in the hands of Dr. John

Clay of England; a claim which has not been corroborated by the experience of other observers. This subject will doubtless sink to its normal level in the present year.

"Incision of the cervix uteri" had its day before the years covered by my tables, and is fast sinking into oblivion.

Laceration of the cervix uteri and its end, by the simple and admirable operations of Dr. Emmet, is the topic of the day, in this country at any rate, but can scarcely be said to have passed from the state of novelty to that of criticism. It is destined to be a fertile topic for some years to come, when it, too, will be assigned its proper sphere—a very restricted one as I believe—and cease to excite discussion.

The treatment of the "Vomiting in Pregnancy," by dilatation of the cervix uteri, devised by Dr. Copeman, of England, brought this topic forward in 1878 and 1879, as seen by the figures. Its decadence in 1880 is manifest.

The subject of "Lying-in Hospitals" was prolific of discussion in 1878, especially in 1879, but is now on the wane.

*General Conclusions.*—The above quantitive analysis of obstetric and gynæcological literature with regard to nationalities, manifests the prominence of America in this branch of medicine. America contributes more journal articles than any other nation, supports by contributions, both literary and pecuniary, as many special periodicals as France, and twice as many as either England or Germany, and carries on as many special societies as all the other countries of the world together.

England, despite the labors of Wells, Keith, Thornton, Barnes, Duncan, Tait, Leishman and Playfair, is fast losing its pre-eminence in this branch of medicine, and has recently demonstrated its inability to support even one special journal, by the discontinuance of the *Obstetric Journal of Great Britain and Ireland*, on January 1 of the present year.

France is exhibiting an unnatural activity, under special influences already adduced.

Germany holds on the even tenor of its way, while Belgium, Italy, Spain, Denmark, and Russia are awakening to a more active participation in the advance and dissemination of obstetric and gynæcological lore.

I have throughout these pages restricted myself to a quantitative study of the literature. I cannot close without giving in a few words an estimate of the quality of each nation's contributions to the science and practice of gynæcology and obstetrics.

Germany unquestionably advances pure science more than any other nation; the papers in its three journals are the most profound and the most critical.

France manifests a great dearth of original ideas and a most discursive style of discussion, but considerable painstaking historical research. Its journals are prolix and, for the most part, profitless reading, and exceed in number the legitimate demand.

England exhibits a waning interest in this branch of medicine, little originality, but a notable discrimination in adopting new theories and applying them to practice. Its only special journal died at the close of the last year.

To America I have no hesitation in according pre-eminence in this special field. Our countrymen meet the emergencies incident to child-bearing with a quickness of perception and readiness of action rarely seen in other countries. Their ingenuity has led them to devise new operations in gynæcology and to carry their art with brilliant results, so that to-day the practice of that branch has reached a stage here far in advance of other nations. Of course our natural aptitudes lead many of us to overestimate the beneficial results of surgery, but, taken all in all, close observation and study in most of the countries of Europe has confirmed me in the opinion that in obstetrics and gynæcology America leads the world.

